# Reviews

**Published:** 1880-10

**Authors:** 


### Reviews.

---

**The Microscopist.** A Manual of Microscopy and compendium of the Microscopic Sciences, Micro-mineralogy, Micro-chemistry, Biology, Histology, and Practical Medicine. Fourth edition, greatly enlarged, with 252 illustrations. By J. H. WYTHE, A. M., M. D., Professor of Microscopy and Histology, in the Medical College of the Pacific, San Francisco, Cal. Philadelphia: Lindsay & Blakiston, 1880.

We gladly welcome a new edition of this valuable work. It is one of the most complete text books on the subject. The physician will find every necessary fact or principle relating to the microscope, clearly stated and classified; while the use of the instrument in practical medicine receives the largest attention. The chapters on the use of the microscope in pathology, diagnosis, and etiology, which have been added to this edition, largely increase the value of the book to the practitioner of medicine and to many a worker will prove a light in darkness. The illustrations are numerous and good, and, like all of Lindsay & Blakiston's books, the presswork is excellent. The book should be in the hands of every physician who uses a microscope.

---

**The Practitioner's Reference Book.** By RICHARD J. DUNGLISON, A. M., M. D. Second edition, revised and enlarged. Philadelphia: Lindsay & Blakiston, 1880.

The author endeavors to present to the profession, in a very condensed form, important data, gathered from standard authorities and medical periodicals, which can easily be referred to by the busy practitioner in the daily routine of practice. He has collected and conveniently arranged a vast amount of valuable matter, which the text-books fail to regard of sufficient importance for publication. The younger men of the profession will find rules and directions here for emergencies, doses of important drugs with their incompatibles and a great amount of information difficult to obtain without searching a wider range of authorities than is often at hand, while the older physicians can profitably employ their time in scanning its pages, if for no other purpose than to brush away the cobwebs from their opinionated minds on many subjects in regard to which they are often unconsciously ignorant.

Dr. Dunglison furnishes a very clear description of the metric system and its relation to the system now adopted in the United States Pharmacopœia. He also gives practical hints in matters of medical etiquette, baths, examination of urine, poisons, disinfectants, the use of galvanic battery in medicine and surgery, etc. Included in the work are "dietetic rules and precepts" in which the preparation of a suitable dietary for the sick is given with special forms of diet for the diabetic; also rules for testing and disinfecting impure drinking water, how to conduct post-mortem examinations, etc.

It will be seen the author has gathered from a great variety of sources, much useful knowledge which young men absolutely require in the earlier years of practice. We recommend the work as a valuable compend. The profession, appreciating the Medical Dictionary, of which our author is the editor, have in this work a further reason to respect his labors.

---

**The Pathology, Diagnosis and Treatment of Diseases of Women, including the Diagnosis of Pregnancy.** By GRAILY HEWITT, M. D., Lond. F. R. C. P. Third American, from the third London Edition, revised and enlarged. With one hundred and thirty illustration. Philadelphia: Lindsay & Blakiston. 1880.

Dr. Hewitt's work is recognized as authority by the profession of Great Britain, and it has taken rank in this country with such works as Thomas, Emmet, and Byford, in the special department to which it is devoted. The third edition is essentially a new work, and gives the latest views upon diseases of women. Dr. Hewitt is an exponent of the mechanical system of uterine pathology. He embodies here the results of large experience, imparting a clinical character to the work, which renders it all the more valuable.

Upon many of the subjects here treated, the author differs from our American writers, and emphasizes his opinion in very positive language. Especially is this true in regard to uterine flexions. Dr. Hewitt formulates his opinion upon these displacements as follows: "Disease of the uterus, the essence of which is a change of shape," by which he implies that the flexion is the most important element in the matter. In this respect many would disagree with the conclusions he arrives at, while not differing essentially in the measures he adopts to overcome the displacements. The chapter devoted to dysmenorrhœa is very clear and comprehensive, and offers the best exposition of this painful complication of the menstrual condition we have seen in any text book. The author also devotes a chapter to "Nervous disorders referable to the uterus," in which he takes occasion to direct attention to flexion as a causative element in the production of these derangements.

Indeed the work is replete with the most advanced opinions and suggestions on all the subjects of which it treats, and may well be accepted as a valuable contribution to the literature of the important class of diseases to which it is devoted. It is a gratifying evidence of the study now directed to diseases of women, when in a few years, our author across the water, with Emmet, and Thomas, and Goodell, on this continent, have issued from the press, these scholarly contributions to medical science.

---

**Sea-air and Sea-bathing.** By JOHN H. PACKARD, M. D., Surgeon to the Episcopal Hospital, Philadelphia. Presley Blackiston, 1012 Walnut street. 1880. Buffalo: Peter Paul & Bro.

This is number eleven of the American Health Primer series, and contains many suggestions in regard to the use of sea-water, as a hygienic measure, which will be profitable to those who seek the sea-shore for restoration of impaired health. It is a capital little work which we take pleasure in recommending.

**Fracture of the Patella.** A study of one hundred and twenty-seven cases. By FRANK H. HAMILTON, M. D. New York: Chas. L. Birmingham & Co., 1880.

The distinguished writer has in a neat little book presented his experience of fracture of the patella, based upon the study of 127 cases, of which 54 have come under his own observation, the rest are taken from the records of Bellevue hospital. Every case is reported separately; cause, treatment and result being noticed, and at last a summary of all the cases is given. The different methods of treatment and their value are fully explained, and altogether it sustains the high reputation of the author.

---

**Naso-Pharyngeal Catarrh.** By MARTIN F. COOMES, M. D., Professor of Physiology, etc., in the Kentucky School of Medicine, Louisville, Ky. Bradley & Gilbert, Publishers. 1880.

Any physician, who has had much experience with that bugbear, nasal catarrh, will appreciate this monograph. It goes thoroughly over the ground, considering first, the anatomy, examination and general medication, and then the different forms of catarrh, with their symptoms and special modes of treatment. The book, even if it does not offer anything new, will be welcomed by the general practitioners, for whom it is written.

---

**The Medical Register of New York, New Jersey and Connecticut.** WILLIAM T. WHITE, Editor. Putnam's Sons, Publishers.

If this were only a list of physicians, it would even then be a convenient book for any practitioner. It contains in addition, however, a number of facts relating to the profession which one has frequent occasion to refer to. It gives a list of officers of the various national medical associations, mentioning also the time and place of meeting of the numerous medical associations, in each of these states. Few books, indeed, are as full of information in regard to doctors as a class, as is this small one by Dr. White of New York.

**The Student's Dose Book and Anatomist.** By C. HENRI LEONARD, A. M., M. D., Professor of Medicine and Surgery, Diseases of Woman and Clinical Gynecology, Michigan College of Medicine. 25th thousand. Price $1.00. Detroit.

That this little book has reached so large a circulation is evidence of merit. It contains a wonderful amount of information condensed into so small a compass, that the student may readily carry it in his pocket. It will be found very convenient for memorizing doses and important points in anatomy.

---

**A Treatise on Common Forms of Functional Nervous Diseases.** By L. PUTZEL, M. D.

William Wood & Co., here offer to the public another volume of the "Library of Standard Medical Authors." A great deal of credit is due to that enterprising firm for thus presenting so much valuable reading matter at such a reasonable rate; and, doubtless, this book, like others of the series, will have a large sale. It is virtually a series of monographs on chorea, epilepsy, neuralgia, and on peripheral paralysis, each one of which, with its varieties and subdivisions, is taken up and treated in detail by a writer particularly qualified for the work he has undertaken.

---

**Therapeutics.** Translated by D. F. LINCOLN, M. D., from the Materia and Medica and Therapeutics of A. TROSSEAU, M. D., Professor of Therapeutics of the Faculty of Medicine of Paris, Physician to l'Hotel Dieu, etc., etc. H. PIDOUX, M. D., Member of the Academy of Medicine, Paris, etc., etc., and CONSTANTINE PAUL, M. D., Adjunct Professor of the Faculty of Paris, Physician to the St. Antoine Hospital, etc. Ninth French Edition, Revised and Edited. Vol. 1 and 2. New York: William Wood & Co.

Trosseau's great work on Therapeutics makes three volumes of Wood's Library, and will prove a most valuable addition to the libraries of American physicians. Heretofore, though often quoted, it has been accessible only in the French. Many of the remedies commended by him have been little resorted to by physicians in this country, but their use in Europe is based upon a mass of experience, which entitles them to careful attention. We hope that all our readers are subscribers for "The Library." Every volume of the series published has been of notable value.

---

**American Newspaper Directory**. New York: GEO. P. ROWELL & Co.

During the past year a prospectus of this work was sent us with a request for information. We supposed it to be chiefly an advertising "dodge," and paid no attention to the request. We now find on our table a handsome volume of over one thousand pages, containing a great many advertisements, it is true, but also apparently accurate lists of all the newspapers and periodicals published in the United States, Territories and the Dominion of Canada. The work shows a vast amount of care and labor in its preparation, and we suppose that the information furnished is fairly reliable. It is sufficiently annoying to find THE JOURNAL credited with about half its present circulation, and still edited (according to the Directory) by Dr. Miner; but we cannot fairly blame the publishers for the error, as we failed to give them the information asked.

